# Hepatocyte DDX3X protects against drug-induced acute liver injury via controlling stress granule formation and oxidative stress

**DOI:** 10.1038/s41419-023-05913-x

**Published:** 2023-07-06

**Authors:** Tingting Luo, Suzhen Yang, Tianming Zhao, Hanlong Zhu, Chunyan Chen, Xiaoxiao Shi, Di Chen, Kai Wang, Kang Jiang, Dan Xu, Ming Cheng, Juan Li, Wenting Li, Weijun Xu, Lin Zhou, Mingzuo Jiang, Bing Xu

**Affiliations:** 1 Department of Gastroenterology, Nanjing Drum Tower Hospital, Affiliated Hospital of Medical School, Nanjing University, Jiangsu Nanjing, 210008 China; 2 Department of Gastroenterology and Hepatology, Jinling Hospital, Affiliated Hospital of Medical School, Nanjing University, Jiangsu Nanjing, 210002 China; 3 Key Laboratory of Resource Biology and Biotechnology in Western China, Ministry of Education, School of Medicine, Northwest University, Shaanxi Xi’an, 710069 China; 4 Department of Gastroenterology, Nanjing Drum Tower Hospital, Chinese Academy of Medical Science & Peking Union Medical College, Nanjing, 210008 Jiangsu China; 5 State Key Laboratory of Cancer Biology, National Clinical Research Center for Digestive Diseases and Xijing Hospital of Digestive Diseases, the Air-Force Military Medical University, Shaanxi Xi’an, 710032 China; 6 Department of Gastroenterology, 949th Hospital of Chinese People’s Liberation Army, Xinjiang Altay, 836500 China

**Keywords:** Cell death, Molecular biology

## Abstract

Drug-induced liver injury (DILI) is the leading cause of acute liver failure (ALF). Continuous and prolonged hepatic cellular oxidative stress and liver inflammatory stimuli are key signatures of DILI. DEAD-box helicase 3, X-linked (DDX3X) is a central regulator in pro-survival stress granule (SG) assembly in response to stress signals. However, the role of DDX3X in DILI remains unknown. Herein, we characterized the hepatocyte-specific role of DDX3X in DILI. Human liver tissues of DILI patients and control subjects were used to evaluate DDX3X expression. APAP, CCl4 and TAA models of DILI were established and compared between hepatocyte-specific DDX3X knockout (DDX3X^Δhep^) and wild-type control (DDX3X^fl/fl^) mice. Hepatic expression of DDX3X was significantly decreased in the pathogenesis of DILI compared with controls in human and mice. Compared to DDX3X^fl/fl^ mice, DDX3X^Δhep^ mice developed significant liver injury in multiple DILI models. DDX3X deficiency aggravates APAP induced oxidative stress and hepatocyte death by affecting the pro-survival stress granule (SG) assembly. Moreover, DDX3X deficiency induces inflammatory responses and causes pronounced macrophage infiltration. The use of targeted DDX3X drug maybe promising for the treatment of DILI in human.

## Introduction

The liver is a major organ for drug metabolism and elimination [1]. Drug-induced liver injury (DILI) has become a leading cause of acute liver failure (ALF) and transplantation [2]. Acetaminophen (APAP) is the most well-studies drug which overdose induces hepatotoxicity and ALF [3, 4]. APAP is metabolized by cytochrome P450 isoforms into N-acetylbenzoquinoneimine (NAPQI) [5]. Excess NAPQI formation due to APAP overdose overwhelms the antioxidant glutathione (GSH) reserves of liver, causing accumulating of cytotoxic adducts and subsequent hepatotoxicity [6, 7]. Apart from APAP, established mice models also utilizes carbon tetrachloride (CCl4) or thioacetamide (TAA) to interpret the key aspects of DILI [7, 8]. Regardless of the etiology, profound hepatic cellular oxidative stress and persistent liver inflammation are key signatures of DILI, which aggravating hepatocyte injury and hepatocellular cell death [9–11]. However, the key molecular mechanisms that interprets these stress responses in the pathogenesis of DILI remains largely unknown.

The DEAD-box helicase 3, X-linked (DDX3X), a DEAD-box family member and helicase, has been implicated in human health and disease, regulates viral replication, innate immune response, and translational reprogramming [12–14]. DDX3X is a key molecule govern cell fate when exposed to stress stimuli by controlling the formation of pro-survival stress granule (SG) and pro-death NLRP3 activation [15, 16]. SGs are cytoplasmic messenger ribonucleoprotein (mRNP) aggregates that assemble during cellular stress (such as hypoxia, heatshock and oxidative stress) [17, 18]. Evidence of the DDX3X interacts with SG in hepatitis C virus infection has been documented [19], suggesting that DDX3X-mediated SG formation may be important for liver pathogenesis. However, the possible role of DDX3X in DILI is still unknown.

In this study, we used conditional hepatocyte DDX3X knockout mice (DDX3X^Δhep^) and DDX3X control mice (DDX3X^fl/fl^) to explore the hepatocyte-specific role of DDX3X in DILI. Our results demonstrated that downregulation of DDX3X is found in human and multiple mice models of DILI. DDX3X deficiency impairs the capacity of SG assembly, leading to persistent and profound hepatic oxidative stress, cell death and subsequent inflammation. Therefore, targeting DDX3X may provide a novel strategy for treating drug-induced hepatotoxicity.

## Materials and methods

### Human liver tissues

Human DILI liver tissues (*n* = 16) were obtained from percutaneous liver biopsy from the Nanjing Drum Tower Hospital, Affiliated Hospital of Medical School, Nanjing University. Control liver samples (*n* = 16) originated from patients with hepatic hemangioma. Histological assessments were determined by two pathologists in a double-blind manner. All patients provided written informed consent. The study was approved by the Institutional Ethics Committee of Nanjing Drum Tower Hospital.

### Mice and treatments

All animal studies were performed in accordance with the guidelines of the Institutional Animal Use and the Animal Experimentation Ethics Committee at the Northwest University. Mice were maintained in ventilated cages under 12 h light/dark cycles. The DDX3X^fl/fl^ mice (C57BL/6 strain) was generated as previously described [15]. DDX3X^∆hep^ mice were established by crossing DDX3X^fl/fl^ mice with Alb-cre mice (C57BL/6 strain). Sex-matched mice at 8 weeks of age were used for all the experiments. To generate drug induced liver injury, DDX3X^∆hep^ mice and DDX3X^fl/fl^ littermates were fasted overnight before intraperitoneally injected with APAP (350 mg/kg) or CCL4 (1 ml/kg) or TAA (300 mg/kg) as previously report [7, 8]. Serum and liver tissues were collected.

### Statistical analysis

All experiments were repeated at least three times with consistent results. Statistical analysis was performed using the statistical software package SPSS 13.0 (SPSS, Chicago, IL, USA) and quantitative data were expressed as the Median (25th, 75th percentiles) or mean ± SD. All tests of significance were two-sided and *p*-values < 0.05 were considered statistically significant.

Additional material and methods are reported in the Supplementary materials.

## Results

### DDX3X expression is downregulated in human and mice with drug-induced liver injury (DILI)

**Fig. 1:**
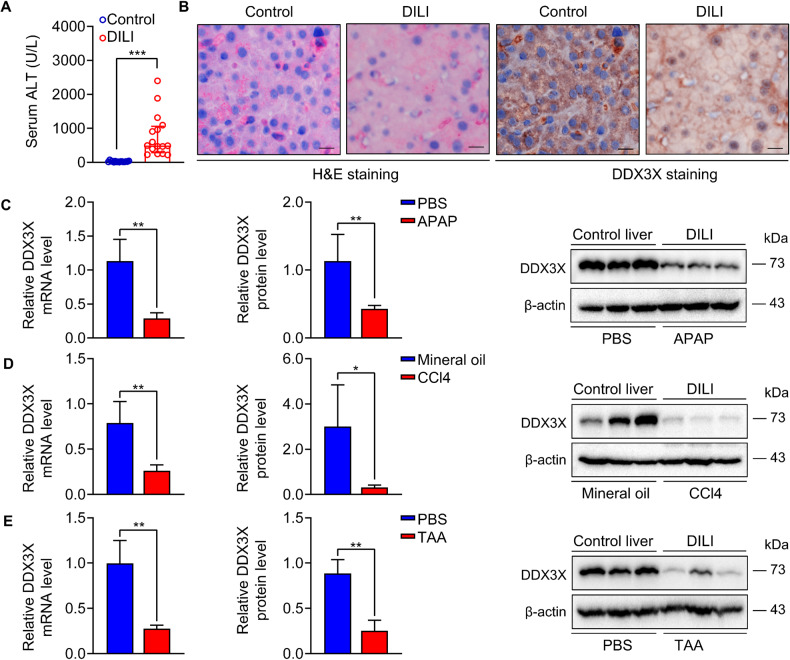
DDX3X expression is decreased in the livers of humans and mice with DILI. **A** Serum levels of ALT of control subjects and patients with DILI. Data are expressed as the Median (25th, 75th percentiles), ***p < 0.001. **B** Representative H&E and IHC sections of human liver tissues. Scale bars, 10 µm; **C**–**E** mRNA and protein expression of DDX3X in multiple DILI mouse models treated with APAP, CCL4, or TAA. Data are expressed as mean ± SD, *n* = 3-6/group. *p < 0.05, **p < 0.01.

To elucidate the role of DDX3X in DILI, we examined DDX3X expression in human liver sections with DILI. All patients with DILI gained higher serum levels of alanine aminotransferase (ALT) and aspartate transaminase (AST) compared with controls (Fig. 1A and Supplementary Table. S1). Immunohistochemical (IHC) staining revealed that DDX3X is widely expressed in hepatocytes and non-parenchymal cells of liver with control subjects, and hepatic expression of DDX3X was significantly decreased in the pathogenesis of DILI (Fig. 1B). Consistent with human data, downregulation of DDX3X was also found in multiple mice models of DILI (Fig. 1C–E), suggesting that DDX3X downregulation is involved in the pathogenesis of DILI in human and mice.

### Hepatocyte DDX3X protects against APAP induced liver injury

**Fig. 2:**
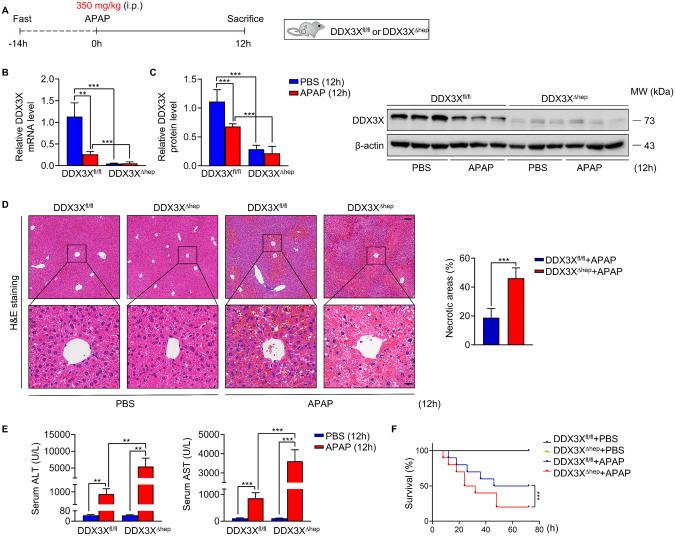
Deficiency of hepatocyte DDX3X exacerbate APAP induced DILI in mice. **A** Schematic diagram of APAP induced DILI model; **B**, **C** mRNA and protein expression of DDX3X in mice treated with PBS or APAP for 12 h. *n* = 3/group; **D** Histological evaluation of liver sections from DDX3X^fl/fl^ and DDX3X^Δhep^ mice treated with APAP or PBS for 12 h, respectively. Scale bars, 100 µm (top), 20 µm (bottom). *n* = 6/group; **E** Serum levels of ALT and AST of DDX3X^fl/fl^ and DDX3X^Δhep^ mice with APAP induction. *n* = 6/group; **F** Survival assay of DDX3X^fl/fl^ and DDX3X^Δhep^ mice treated with APAP or PBS for 72 h, respectively. *n* = 10/group. Data are expressed as mean ± SD. **p < 0.01.

Given that hepatocyte is the major cell type involved in DILI, we investigated the role of hepatocyte DDX3X in the development of DILI by APAP treatment for 12 h in DDX3X^fl/fl^ and DDX3X^∆hep^ mice, respectively (Fig. 2A). DDX3X mRNA and protein were readily expressed in DDX3X^fl/fl^ mice, but not in DDX3X^∆hep^ mice (Fig. 2B, C). Histological analysis by H&E staining showed more severe liver injury in DDX3X^∆hep^ mice compared with DDX3X^fl/fl^ controls induced with APAP (Fig. 2D). Consistently, ablation DDX3X causes elevated serum levels of ALT and AST (Fig. 2E). Corresponding to the protective role of hepatocyte DDX3X in DILI, survival assay showed significantly improved survival of DDX3X^fl/fl^ mice compared to DDX3X^∆hep^ mice (Fig. 2F), indicating that DDX3X downregulation in hepatocytes is a major cause of liver injury in APAP induced mice.

### Hepatocyte DDX3X protects against CCl4 or TAA induced liver injury

**Fig. 3:**
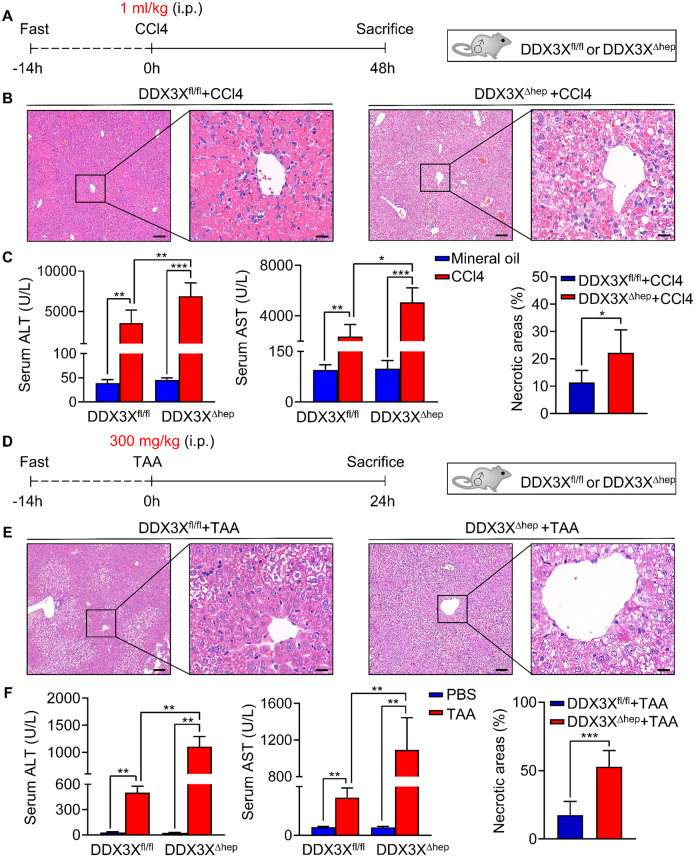
Deficiency of hepatocyte DDX3X exacerbate CCl4 and TAA induced DILI in mice. **A** Schematic diagram of CCl4 induced DILI model; **B** histological evaluation of liver sections from DDX3X^fl/fl^ and DDX3X^Δhep^ mice treated with CCl4 for 48 h, respectively. Scale bars, 100 µm (left), 20 µm (right); **C** serum levels of ALT and AST of DDX3X^fl/fl^ and DDX3X^Δhep^ mice with CCl4 induction; **D** schematic diagram of TAA induced DILI model; **E** Histological evaluation of liver sections from DDX3X^fl/fl^ and DDX3X^Δhep^ mice treated with TAA for 24 h, respectively. Scale bars, 100 µm (left), 20 µm (right); **F** serum levels of ALT and AST of DDX3X^fl/fl^ and DDX3X^Δhep^ mice with TAA induction. Data are expressed as mean ± SD, *n* = 6/group. *p < 0.05, **p < 0.01, ***p < 0.001.

The effects of hepatocyte DDX3X on DILI were further validated in CCl4 and TAA induced DILI model (Fig. 3A and D). Extensively liver injury, elevated ALT and AST levels were presented in DDX3X^∆hep^ mice compared with DDX3X^fl/fl^ controls after CCl4 induction (Fig. 3B, C). Consistently, DDX3X^∆hep^ mice with TAA induction also showed extensive liver injury, increased ALT and AST levels compared with DDX3X^fl/fl^ controls with TAA treatment (Fig. 3E, F), further validating that hepatocyte DDX3X plays a protective role against DILI.

### Hepatocytes DDX3X is required for stress granules formation and cell survival in APAP-induced liver injury

**Fig. 4:**
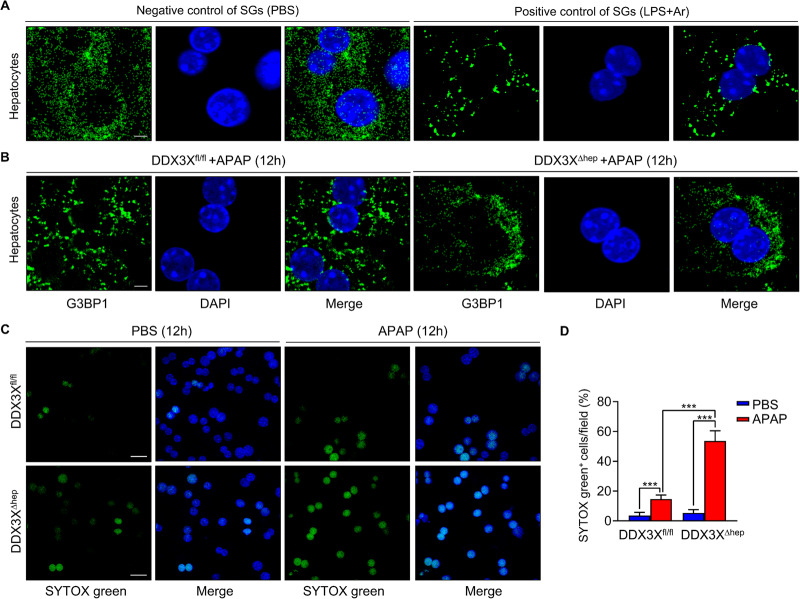
Hepatocytes DDX3X is required for stress granules formation and cell survival in APAP-induced liver injury. **A** Negative control of primary hepatocytes were treated with PBS; and positive control of SG in primary hepatocytes were first primed with LPS for 4 h, then induced by 50 μM sodium (meta) arsenite for 30 min; **B** representative IF stained sections of G3BP1 (a marker of SGs) in primary hepatocytes isolated from DDX3X^fl/fl^ or DDX3X^Δhep^ mice with in vitro stimulation of APAP for 12 h. Scale bars, 10 µm; **C** representative IF stained sections of SYTOX green in primary hepatocytes isolated from DDX3X^fl/fl^ or DDX3X^Δhep^ mice with in vitro stimulation of APAP or PBS for 12 h, respectively. Scale bars, 20 µm; **D** Quantitative data of SYTOX green^+^ cells of primary hepatocytes; Data are expressed as mean ± SD, *n* = 3/group. ***p < 0.001.

DDX3X is a key regulator driving pro-survival SGs assembly to respond to changes in homeostatic flux [15, 20]. To demonstrate whether the deteriorated liver injury of DDX3X^∆hep^ mice is due to the impairment of SGs formation, we examined the role of DDX3X in hepatocyte SG assembly under APAP treatment. Confocal microscopy imaging of G3BP1 (a marker of SGs) in DDX3X^fl/fl^ hepatocytes showed obviously SG assembly after APAP induction (Fig. 4A, B). However, DDX3X^Δhep^ hepatocytes gained less SG formation compared to DDX3X^fl/fl^ hepatocytes (Fig. 4A, B). In line with that, more SYTOX green^+^ hepatocytes were found in APAP-treated DDX3X^∆hep^ mice compared with APAP-treated DDX3X^fl/fl^ mice (Fig. 4C, D), suggesting that hepatocyte DDX3X deletion induces severe DILI may at least partly due to deficiency of pro-survival SG formation, thus impairing the capacity of hepatocytes to respond to stress stimuli.

### Hepatocyte DDX3X ablation aggravated oxidative stress

**Fig. 5:**
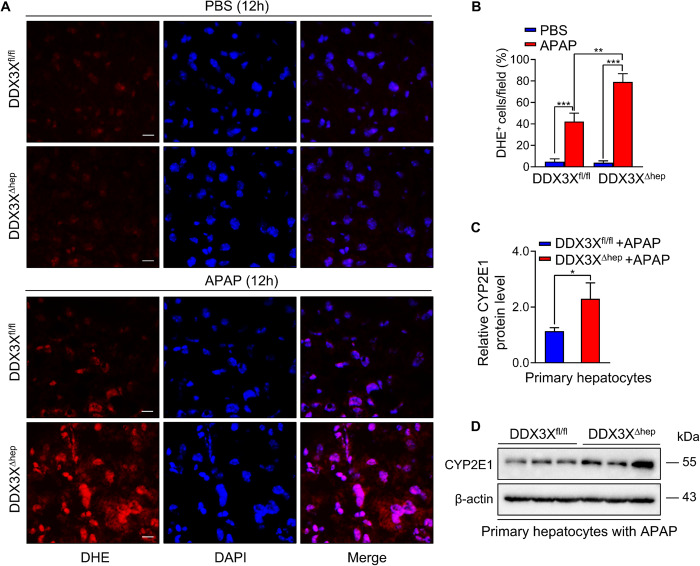
Hepatocyte DDX3X ablation induces oxidative stress in DILI. **A** Representative DHE stained liver sections of DDX3X^fl/fl^ and DDX3X^Δhep^ mice treated with APAP or PBS. Scale bars, 25 µm; **B** calculation of DHE^+^ cells of liver sections; **C**, **D** protein expression of CYP2E1 in primary hepatocytes treated with APAP for 12 h. Data are expressed as mean ± SD, *n* = 3/group. *p < 0.05, **p < 0.01, ***p < 0.001.

Extensive production of reactive oxygen species (ROS) during drug metabolism is a prominent feature of APAP-induced hepatotoxicity [21, 22]. SGs formation is a key antioxidant process to protects cellular homeostasis in response to oxidative stress [23]. To characterize whether the deteriorated liver injury caused by DDX3X deficiency is due to impairment of antioxidant process, we examined the ROS production. Dihydroethidium (DHE) staining revealed that APAP-treated DDX3X^∆hep^ mice displayed more extensive ROS production compared with APAP-treated DDX3X^fl/fl^ mice (Fig. 5A, B). In agreement with the ROS induction, higher level of APAP-metabolizing enzyme cytochromeP450, family2, subfamilyE, polypeptide1(CYP2E1) was seen in hepatocytes isolated from DDX3X^∆hep^ mice compared with hepatocytes isolated from DDX3X^fl/fl^ mice in APAP medium (Fig. 5C, D). Taken together, these results indicates that hepatocyte DDX3X deletion aggravated oxidative stress in APAP-induced liver injury.

### Hepatic DDX3X deletion exacerbates macrophage infiltration and inflammation

**Fig. 6:**
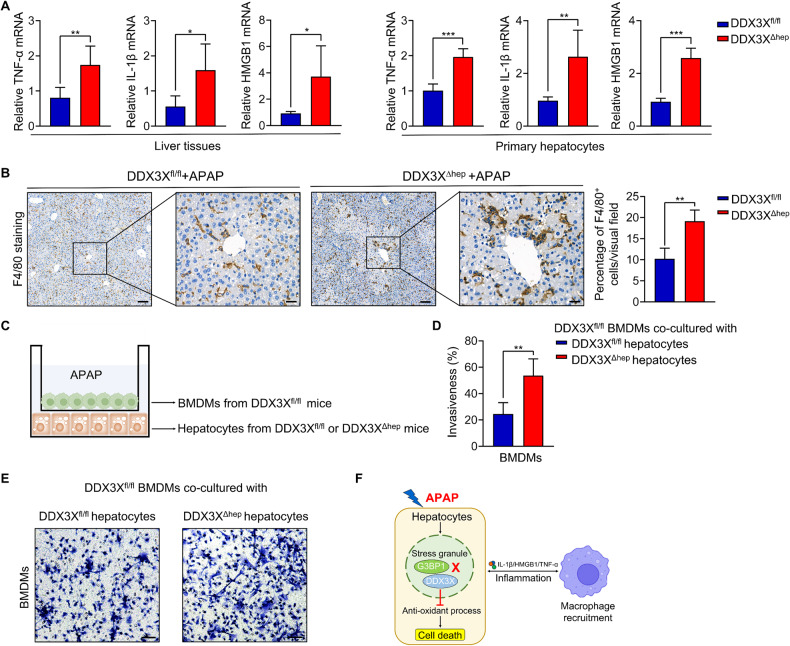
Hepatic DDX3X deletion exacerbates macrophage infiltration and inflammation. **A** mRNA expression levels of IL-1β, HMGB1 and TNF-α in liver tissues and primary hepatocytes of DDX3X^fl/fl^ and DDX3X^Δhep^ mice; **B** Representative IHC stained sections of F4/80 in liver tissues of APAP induced DILI. Scale bars, 100 µm (left), 25 µm (right); **(C)** Schematic diagram of DDX3X^fl/fl^ macrophage co-cultured with DDX3X^fl/fl^ or DDX3X^Δhep^ hepatocytes in APAP medium; **D, E** Numbers of infiltrated macrophages were evaluated by co-culture system. Scale bars, 100 µm; **F** Schematic diagram of DDX3X in DILI. DDX3X knockout in the hepatocytes of mice significantly exacerbated histological severity in multiple DILI murine models. This effect is through regulation of SG formation to protect hepatocytes from oxidative stress and macrophage inflammation. Data are expressed as mean ± SD, *n* = 3-6/group. *p < 0.05, **p < 0.01, ***p < 0.001.

To explain the pronounced inflammation in the livers of DDX3X^Δhep^ mice, we evaluated hepatic mRNA expression of pro-inflammatory cytokines (including TNF-α, IL-1β and HMGB1) from liver tissues of DDX3X^Δhep^ mice, and found these cytokines were all significantly higher in DDX3X^Δhep^ mice than in DDX3X^fl/fl^ mice with APAP treatment (Fig. 6A). Consistently, higher mRNA expression of TNF-α, IL-1β and HMGB1 were also found from primary hepatocytes isolated from DDX3X^Δhep^ mice compared with DDX3X^fl/fl^ mice (Fig. 6B). Considering that upregulation of these cytokines is tightly associated with macrophage-mediated inflammation, we evaluated hepatic macrophage infiltration by F4/80 immunostaining. DDX3X^Δhep^ mice showed numerous and obvious macrophage infiltration in the livers compared with DDX3X^fl/fl^ mice in APAP-induced DILI model (Fig. 6C). In addition, DDX3X^fl/fl^ macrophages co-cultured with DDX3X^Δhep^ hepatocytes showed increased numbers of infiltrated macrophage compared to DDX3X^fl/fl^ macrophages cocultured with DDX3X^fl/fl^ hepatocytes in vitro (Fig. 6D, E). Taken together, these data demonstrated that loss of DDX3X in hepatocytes induces macrophage infiltration, thus facilitating the inflammatory responses in APAP-induced DILI.

## Discussion

The first novel finding in present study is that DDX3X^Δhep^ mice administrated with APAP, CCl4 or TAA showed seriously hepatotoxicity compared with DDX3X^fl/fl^ mice induced with same drugs. These findings were corroborated by aggregated liver histology and elevated serum ALT and AST. Moreover, DDX3X deletion was associated with a significant induction of oxidative stress and cell death. This change was tightly associated with impaired SG formation of hepatocytes, thus losing protection from ROS insults induced by APAP overdose. In addition, seriously hepatotoxicity caused by DDX3X deficiency induces pro-inflammatory cytokines (such as HMGB1, IL-1β and TNF-α) production and recruitments macrophage infiltration, which perpetuate of liver injury.

Given the downregulation of DDX3X in human and mice liver of DILI, we addressed its importance by generating hepatocyte specific DDX3X knockout mice [15]. The DDX3X^Δhep^ mice demonstrated no obvious liver phenotype under basal conditions compared with DDX3X^fl/fl^ mice. However, DDX3X^Δhep^ mice displayed deteriorate liver injury and pronounced liver inflammation compared with DDX3X^fl/fl^ mice under treatment of series drugs. Considering that APAP is the most widely used drug to explore the key aspects of DILI, we select APAP to explain the molecular mechanisms by which DDX3X exerts its functions in DILI. SGs formation is a major antioxidant process to protects cellular homeostasis in response to oxidative stress [23]. As a key decision maker governing the cell fate, DDX3X plays an important role to form the pro-survival SG [15]. We found that under APAP treatment, DDX3X^∆hep^ hepatocyte displayed less SG assumable compared with DDX3X^fl/fl^ hepatocyte. Moreover, DDX3X^∆hep^ hepatocyte is more prone to die compared with APAP-treated DDX3X^fl/fl^ hepatocyte, suggesting that hepatocyte DDX3X deletion induces severe DILI may at least partly due to impairing the capacity of hepatocytes SGs formation to response to stress stimuli. In addition, compared with APAP-treated DDX3X^fl/fl^ mice, more pronounced ROS production and elevated level of APAP-metabolizing enzyme CYP2E1 were also found in the liver of DDX3X^∆hep^ mice, implying that loss of DDX3X make hepatocyte more sensitive to oxidative stress. Moreover, we noticed an increased expression of pro-inflammatory cytokines and infiltration of macrophage of DDX3X^∆hep^ mice compared with DDX3X^fl/fl^ mice, indicating that lack of DDX3X might enhance the hepatocyte production of pro-inflammatory cytokines and macrophage infiltration, thus facilitating the inflammatory responses in APAP-induced DILI.

In conclusion, these observations demonstrate for the first time that DDX3X plays an essential role in the development of DILI. Further, the mechanism of this effect is through regulation of SG formation to protect hepatocytes from oxidative stress and macrophage inflammation. The use of targeted DDX3X drug maybe promising for the treatment of DILI in human.

## Supplementary information

Supplementary information

Original data

aj-checklist

## Data Availability

The data that support the findings of this study are available from the corresponding author upon reasonable request.
