# Supplementary material for: Hepatocyte DDX3X protects against drug-induced acute liver injury via controlling stress granule formation and oxidative stress
Source: Cell Death Dis. 2023 Jul 6;14(7):400. doi: 10.1038/s41419-023-05913-x (PMC10322869; doi:10.1038/s41419-023-05913-x)
Supplement: Supplementary file 1 — Supplementary information [file 41419_2023_5913_MOESM1_ESM.docx]

**Hepatocyte DDX3X protects against drug-induced acute liver injury via controlling stress granule formation and oxidative stress**

Tingting Luo, Suzhen Yang, Tianming Zhao, Hanlong Zhu, Chunyan Chen, Xiaoxiao Shi, Di Chen, Kai Wang, Kang Jiang, Dan Xu, Ming Cheng, Juan Li, Wenting Li, Weijun Xu, Lin Zhou, Mingzuo Jiang, Bing Xu

**Table of contents**

Supplementary Materials and Methods ........................................................ 2

Table S1 ....................................................................................................... 5

Table S2 ....................................................................................................... 5

**Supplementary materials and methods**

**Histological examination and biochemical assays**

Liver tissues were fixed with 4% paraformaldehyde overnight and then embedded in paraffin. H&E-stained liver sections were obtained and analyzed by pathologists in a double-blind manner.

Serum levels of alanine aminotransferase (ALT) and aspartate transaminase (AST) were determined by using the ALT and AST assay kit (Rayto, Shenzhen, China).

**Immunohistochemical (IHC) staining**

Immunohistochemical staining was performed according to manufacturer's instruction by using immunohistochemistry assay kit (Beyotime, Shanghai, China). In brief, paraffin-embedded liver sections were deparaffinized and the antigens were retrieved. The slides were covered in 3% hydrogen peroxide (H_2_O_2_) solution to abolish endogenous peroxidase activity, followed by blocked with 5% goat serum. Then the slides were incubated with primary antibodies at 4 °C overnight, followed by incubation with HRP-labeled secondary antibodies for 30 min and stained with DAB solution (Zsbio, Beijing, China) for microscopic observation. Then, the nuclei were counterstained with hematoxylin. Finally, the slides were dehydrated, sealed and photographed. Detail of the primary antibodies used in IHC analysis was shown in ***Table S3.***

**Dihydroethidium (DHE) staining**

Frozen liver sections were fixed with pre-cooled 100% acetone. The detection of superoxide was accomplished via DHE staining using a commercially available kit (D11347, Thermo Fisher, Germany) and following the manufacturer’s protocol.

**SYTOX Green nucleic acid staining**

SYTOX (MX4228, Maokangbio, Shanghai, China) was diluted in DMEM medium to a final concentration of 1μM. Hepatocytes isolated from DDX3X^fl/fl^ or DDX3X^∆hep^ mice were incubated in working solution for 1 hour and washed and fixed in 4% paraformaldehyde for 10 minutes. Then the hepatocytes were stained with DAPI for 20 minutes at room temperature.

**Cell culture and treatment**

Primary hepatocytes were isolated by *in situ* liver perfusion from male DDX3X^fl/fl^ and DDX3X^∆hep^ mice based on a two-step collagenase perfusion technique[^1^](#_ENREF_1). In brief, hepatocytes were dissociated from mice by non-recirculating perfusion with ethylene glycol tetraacetic acid (EGTA) buffer followed by collagenase IV (Sigma-Aldrich, St. Louis, MO, USA) digestion via the portal vein. The isolated cells were then passed through a 70 μm cell filter, followed by centrifuged at 40 x g for 5 min at 4 °C. The hepatocyte pellets were collected and cultured in Dulbecco's modification of Eagle medium (DMEM) medium[^2^](#_ENREF_2). Bone marrow cells were isolated from DDX3X^fl/fl^ mice. The femur and tibia of mice were isolated, the bone marrow cells were flushed out with pre-cooled DMEM medium, passed through a 70µm cell strainer, and centrifuged at 350g for 10 min. Then, the cells were incubated with Red Blood Cell Lysis Buffer (Beyotime, Shanghai, China) for 3 minutes at room temperature to lysate red blood cells. After termination of lysis, the cells were centrifuged at 350g for 10 min. The obtained bone marrow cells were cultured in complete DMEM medium containing 20ng/ml m-CSF (PeproTech, USA). After 3 days, the medium was changed, and the non-adherent cells were discarded, and the culture was continued for another 4 days to obtain differentiated mature bone marrow-derived macrophages (BMDMs).

For the co-culture studies, primary hepatocytes from DDX3X^fl/fl^ or DDX3X^Δhep^ mice were cultured with BMDMs from DDX3X^fl/fl^ mice respectively in the transwell chamber (Corning, Arkansas, USA) under APAP culture medium.

**RNA extraction and real-time qPCR analysis**

Total RNA was extracted with GeneJET RNA Purification Kit (Thermo Fisher, Waltham, MA, USA). The Reverse Transcription Master Kit (Thermo Fisher, Waltham, MA, USA) was used to synthesize cDNA. Real-time qPCR was performed using QuantiNova SYBR Green PCR Kit (QIAGEN, HilxXden, Germany), and relative mRNA expression was calculated using the comparative cycle method (ΔΔCt). β-actin was used as control. Specific primers were listed in ***Supplementary table. S2.***

**Protein extraction and Western bolt assay**

Whole lysates of liver tissues and cells were collected by RIPA lysis buffer containing protease and phosphatase inhibitors (Millipore, Billerica, MA, USA). Supernatant was collected by centrifugation at 12,000 rpm for 15 min at 4 °C. Cell lysates were separated by SDS-PAGE and visualized by UltraSignal hypersensitive ECL chemiluminescence substrate (4A Biotech, Beijing, China). β-actin was used as control. Specific primary antibodies were listed in ***Supplementary table. S3.***

**Supplementary References**

1. Zhang T, Li H, Wang K, et al. Deficiency of CD147 Attenuated Non-alcoholic Steatohepatitis Progression in an NLRP3-Dependent Manner. Front Cell Dev Biol 2020;8:784.

2. Zhang X, Fan L, Wu J, et al. Macrophage p38α promotes nutritional steatohepatitis through M1 polarization. J Hepatol 2019;71:163-174.

**Table. S1** Clinical characteristics of all patients’ population.

|  | Control | DILI |
| --- | --- | --- |
| All | 16 | 16 |
| Male | 10 (62.5) | 5 (31.2) |
| Female | 6 (37.5) | 11 (68.8) |
| Age (year) | 51.0 (36.5-57.3) | 50.0 (34.5-57.0) |
| ALT (U/L) | 24.1 (13.2-32.7) | 492.8 (286.1-1062.0) *** |
| AST (U/L) | 19.0 (13.7-20.9) | 309.0 (192.3-747.2) *** |

***Significant at *p*< 0.001, between control subjects and DILI patients. Data are expressed as the Median (25th, 75th percentiles) or N (%). Mann-Whitney U test were used for continuous factors. Abbreviations: N, number of subjects; ALT, alanine aminotransferase; AST, aspartate transaminase.

**Table S2. Real-time qRT-PCR primer sequences**

| Gene | Forward Primer | Reverse Primer |
| --- | --- | --- |
| Mouse  *Ddx3x*  *Il-1β*  *Tnf-α*  *Hmgb1*  *β-actin* | CTATGCCTCCAAAAGGTGTCCG  TCAGGCAGGCAGTATCACTCATT  CGTGCTCCTCACCCACAC  GCTGACAAGGCTCGTTATGAA CATCCGTAAAGACCTCTATGCCAAC | AGACCCAACTCTTCCTACAGCC  GGAAGGTCCACGGGAAAGA  GGGTTCATACCAGGGTTTGA  CCTTTGATTTTGGGGCGGTA  ATGGAGCCACCGATCCACA |

**Table S3. Antibodies used for IHC and Western blot.**

| Antibody | Company | Catalogue number |
| --- | --- | --- |
| F4/80  CYP2E1  DDX3X  β-actin | abcam  abcam  abcam  Sigma-Aldrich | ab100790  ab28146  ab235940  A1978 |
