# Supplementary material for: Hepatocyte DDX3X protects against drug-induced acute liver injury via controlling stress granule formation and oxidative stress
Source: Cell Death Dis. 2023 Jul 6;14(7):400. doi: 10.1038/s41419-023-05913-x (PMC10322869; doi:10.1038/s41419-023-05913-x)
Supplement: Supplementary file 2 — Original data [file 41419_2023_5913_MOESM2_ESM.docx]

**Hepatocyte DDX3X protects against drug-induced acute liver injury via controlling stress granule formation and oxidative stress**

Tingting Luo, Suzhen Yang, Tianming Zhao, Hanlong Zhu, Chunyan Chen, Xiaoxiao Shi, Di Chen, Kai Wang, Kang Jiang, Dan Xu, Ming Cheng, Juan Li, Wenting Li, Weijun Xu, Lin Zhou, Mingzuo Jiang, Bing Xu

**Table of contents**

Western bolts data ........................................................................................2

**Western bolts data**


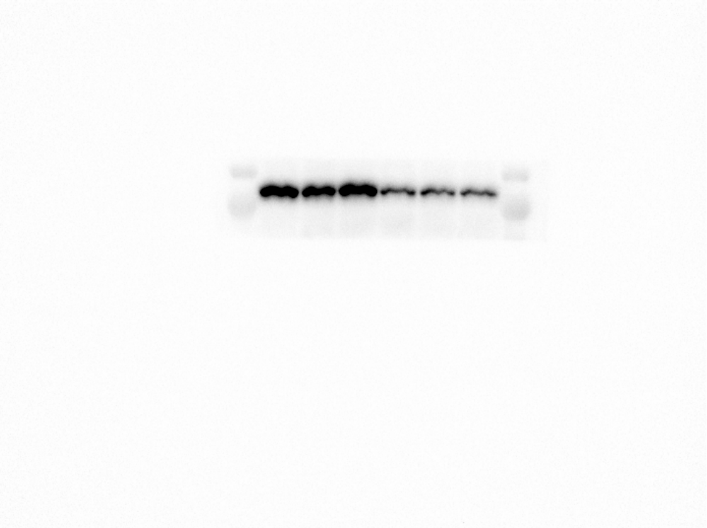


**Figure. 1C DDX3X** (Livers of mice treated with PBS or APAP)


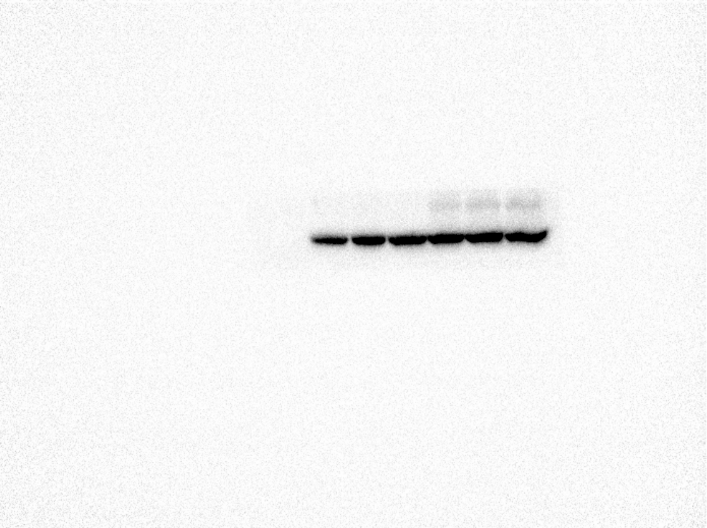


**Figure. 1C β-actin** (Livers of mice treated with PBS or APAP)


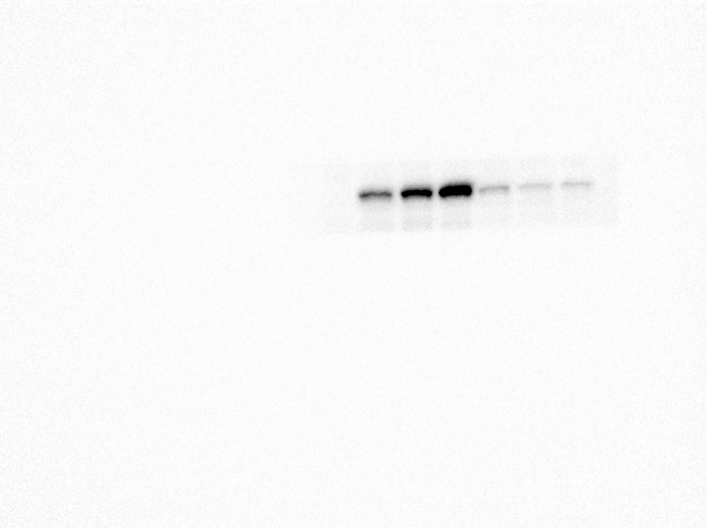


**Figure. 1D DDX3X** (Livers of mice treated with mineral oil or CCL4)


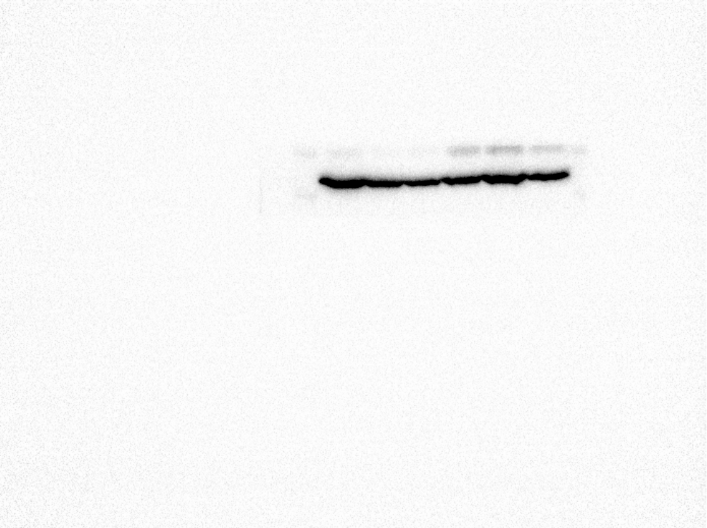


**Figure. 1D β-actin** (Livers of mice treated with mineral oil or CCL4)


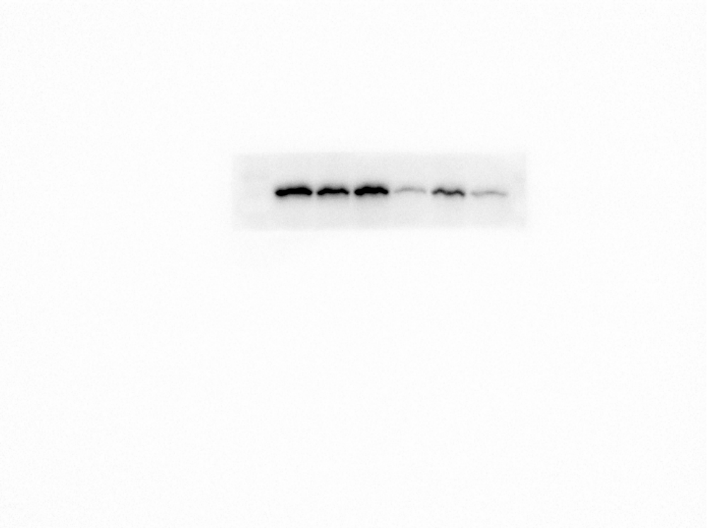


**Figure. 1E DDX3X** (Livers of mice treated with PBS or TAA)


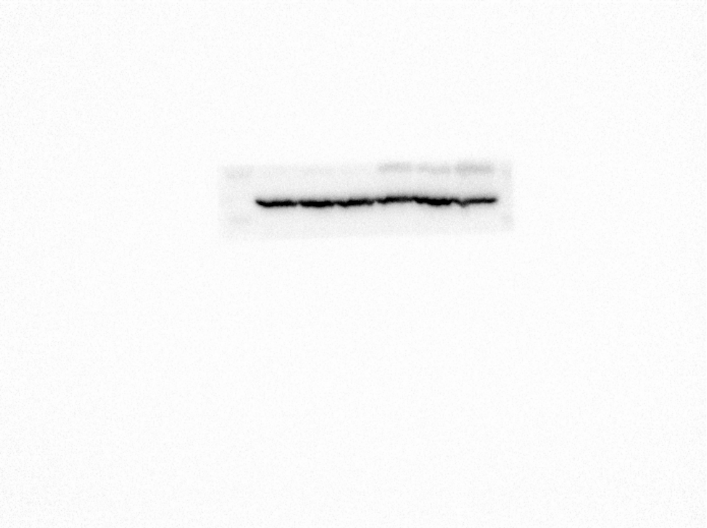


**Figure. 1E β-actin** (Livers of mice treated with PBS or TAA)


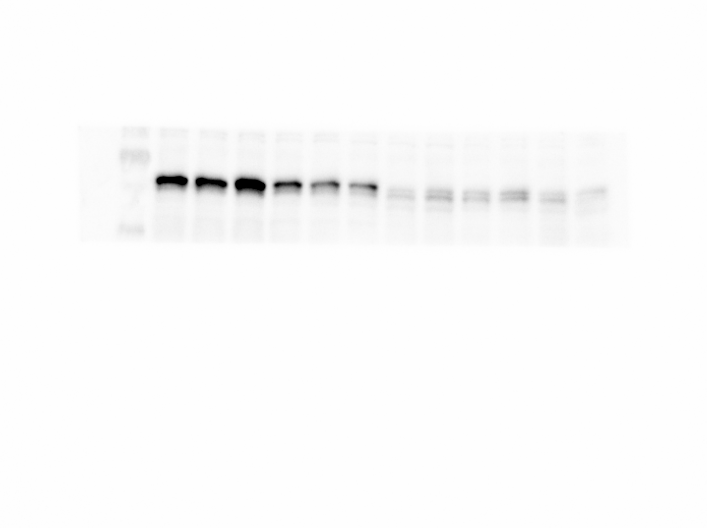


**Figure. 2B DDX3X** (Livers of DDX3X^fl/fl^ mice and DDX3X^∆hep^ mice treated with PBS or APAP)


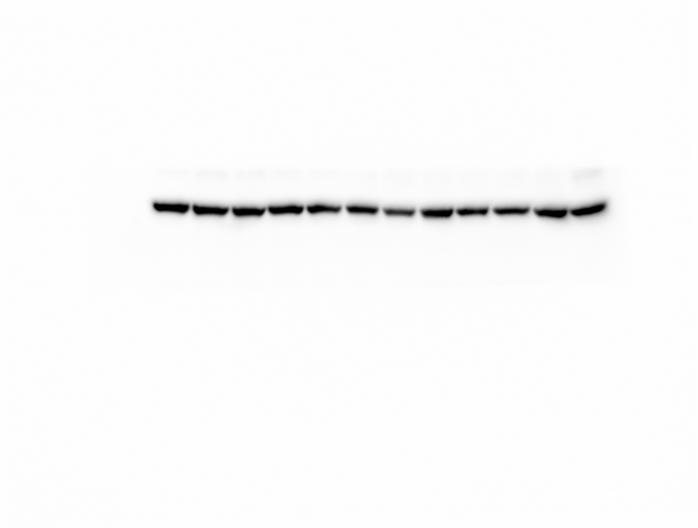


**Figure. 2B β-actin** (Livers of DDX3X^fl/fl^ mice and DDX3X^∆hep^ mice treated with PBS or APAP)


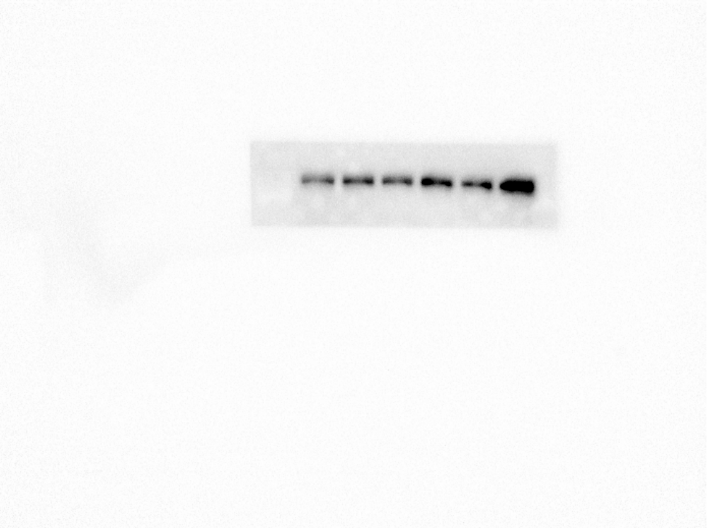


**Figure. 4D CYP2E1** (Hepatocyte of DDX3X^fl/fl^ mice and DDX3X^∆hep^ mice treated with APAP)


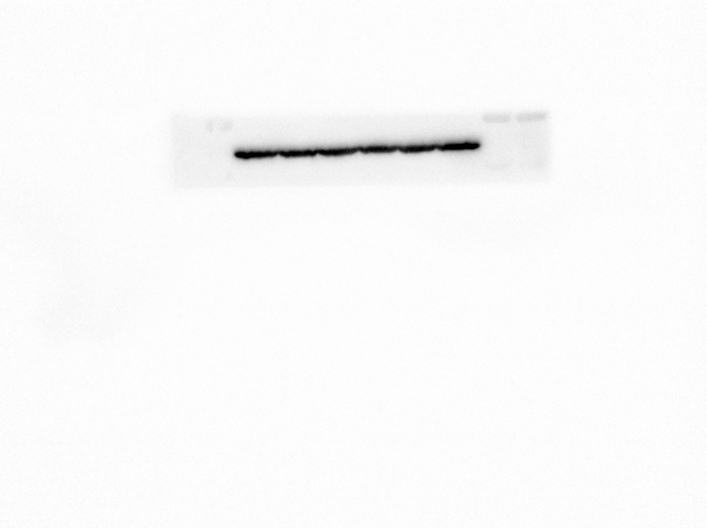


**Figure. 4D β-actin** (Hepatocyte of DDX3X^fl/fl^ mice and DDX3X^∆hep^ mice treated with APAP)
